# Efficacy of intracranial surgical treatments for chronic pain: A systematic review and meta-analysis

**DOI:** 10.1016/j.bas.2026.106157

**Published:** 2026-07-01

**Authors:** Rabea Schmahl, Fátima Ximena Cid-Rodríguez, Rene Marquez-Franco, Petra Heiden, Ricardo Loução, Veerle Visser-Vandewalle, Pablo Andrade

**Affiliations:** a Department of Stereotactic and Functional Neurosurgery, Faculty of Medicine and University Hospital Cologne, University of Cologne, Cologne, Germany; b Postgraduate Department, School of Higher Education in Medicine, National Polytechnic Institute, Mexico City, Mexico; c Department of General Neurosurgery, Faculty of Medicine and University Hospital Cologne, University of Cologne, Cologne, Germany

**Keywords:** Chronic pain, Deep brain stimulation, Cingulotomy, Thalamotomy, Mesencephalotomy

## Abstract

**Introduction:**

Treatment-refractory chronic pain is a prevalent condition affecting a significant portion of the population. Available treatment strategies range from non-invasive to invasive approaches, including several surgical procedures. The most commonly reported intracranial neurosurgical interventions are deep brain stimulation (DBS) and ablative techniques such as cingulotomy, thalamotomy, and mesencephalotomy.

**Research question:**

This study aimed to evaluate the efficacy of different intracranial surgical treatments for refractory chronic pain through a systematic review and meta-analysis, focusing on quantitative trends in clinical pain outcomes.

**Material and methods:**

A systematic literature search was conducted in PubMed according to PRISMA guidelines.

**Results:**

In total, 75 studies including 666 patients that underwent at least one surgical procedure, with nearly half achieving significant pain reduction (>50%). A meta-analysis compared surgical treatments; where mesencephalotomy was excluded due to insufficient data. Pain levels at baseline were compared at four follow-up periods: T1 (≤1 month), T2 (>1–≤6 months), T3 (>6–≤12 months), and T4 (>12 months). Wilcoxon tests demonstrated statistically significant differences between baseline and the latest follow-up for all included methods: DBS: p < 0.001, g = 1.44 85-90%; cingulotomy: p < 0.001, g = 2.6 > 95%; thalamotomy: p < 0.001, g = 0.92 ≈ 74%. Comparisons between follow-up time points were possible only for DBS, which also showed significant differences (p < 0.05).

**Discussion and conclusion:**

Our findings indicate that intracranial surgical procedures for chronic pain are effective/safe and may maintain their clinical benefit for months following treatment. Furthermore, substantial variability in clinical outcomes was observed, even after stratification according to etiology and target groups.

## Highlights

• Systematic review of surgical options for treatment-refractory chronic pain.
• Analysis of 666 patients treated with DBS, cingulotomy, or thalamotomy.
• Approximately 50% of patients achieved >50% pain relief at last follow-up 2.68 years (SD: 3.35 years).
• Significant pain reduction was maintained across all follow-up intervals.
• Quantitative overview of efficacy and safety across surgical treatment modalities.

## 1 Introduction

Treatment-refractory chronic pain is a common disorder that can affect up to half of the adult population (Elliott et al., 1999; Torrance et al., 2006). Approximately 20% of the adult European population suffers from significant treatment-refractory chronic pain, and around 7% to 8% experience intolerable chronic pain with neuropathic features (Torrance et al., 2006; Bouhassira et al., 2008; Breivik et al., 2006). Many of these patients report insufficient pain relief despite pharmacological treatments and may ultimately become candidates for surgical intervention (Ben-Haim et al., 2018).

Surgical techniques for the treatment of treatment-refractory chronic pain have evolved over time. These methods could be broadly divided into two groups: ablative and neuromodulatory procedures. Both approaches have been applied to a variety of peripheral and central anatomical structures, with diverse outcomes reported in the literature (Abdallat et al., 2021; Alamri et al., 2022; Bittar et al., 2005a; Deng et al., 2018; Franzini et al., 2020).

Intracranial surgical methods can likewise be divided into these two categories. The first category that involves ablative procedures, include mesencephalotomy (Amano et al., 1992; Spiegel et al., 1953), thalamotomy (Hanuska et al., 2020; Lovo et al., 2019; Perez de la Torre et al., 2021), and cingulotomy (Deng et al., 2018; Kollenburg et al., 2024; Levi et al., 2019; Wang et al., 2017). The second category, which comprises neuromodulatory procedures refers to deep brain stimulation (DBS) targeting different brain regions (Ben-Haim et al., 2018).

Among the ablative procedures, cingulotomy remains one of the most frequently reported lesioning technique in the literature (Deng et al., 2018, 2019; Kollenburg et al., 2024; Wang et al., 2017; Castillo Rangel et al., 2023; Cohen et al., 1999, 2001; Pillay et al., 1992; Wilkinson et al., 1999). It was developed in the early 1960s to replace frontal lobotomy, primarily used to treat severe, intractable psychiatric disorders, ranging from chronic pain syndromes (including thalamic pain) to severe obsessive-compulsive and panic disorders, anxiety, psychotic depression, and schizophrenia (Faria, 2013). Another well-established lesioning procedure is thalamotomy, which was introduced in the late 1940s for the treatment of pain (Gallay et al., 2023). One notable example is lesioning of the medial nucleus, which disrupts transmission of the affective-motivational component of pain, making it more bearable (Dougherty et al., 2001; Hariz et al., 1995; Jeanmonod and Morel, 2009; Mark et al., 1960; Tasker, 1990; Tasker and Burchiel, 2002). In 1942, Walker et al. demonstrated that the posterior mesencephalon represented the safest and most effective region for accessing the spinothalamic tracts, establishing mesencephalotomy as one of the earliest surgical interventions for chronic pain (Lages et al., 2019). Within the mesencephalon, lesions can be created between the laterally located direct spinothalamic and quintothalamic pathways and the more medially situated diffuse ascending pathways. This approach may provide relief from central or malignant pain without causing substantial sensory deficit (Shieff et al., 1990). Some of the earliest reports of this procedure date back to 1953, when it was used to treat cancer-related pain.

DBS has been used as a reversible method to treat chronic pain since the early 1970s (Bittar et al., 2005a; Hamani et al., 2006; Levy et al., 1987). Various targets have been investigated, including the ventral posteromedial (Vpm), ventral posterolateral (Vpl), centromedian-parafascicular (CM-Pf), ventral anterior (VA) and ventral lateral (VL) thalamic nuclei, the periaqueductal/periventricular gray matter (PAG/PVG), and the anterior cingulate cortex (ACC) (Ben-Haim et al., 2018; Abdallat et al., 2021; Abreu et al., 2022; Boccard et al., 2014a). The ACC constitutes the frontal part of the cingulate cortex (Vogt, 2005) and plays a central role in pain processing (Wang et al., 2024; Xiao et al., 2018). Neurons within the ACC respond to both noxious and non-noxious mechanical or thermal somatosensory stimuli (Hutchison et al., 1999; Koga et al., 2010). Furthermore, neuropathic pain has been associated with mechanisms underlying the expression of long-term potentiation (LTP) within the ACC (Bliss et al., 2016).

Although the literature describes these methods extensively and several reviews have been published (Alamri et al., 2022; Shieff et al., 1990; Allam et al., 2022; Sharim et al., 2016), direct comparisons across all approaches are lacking. Furthermore, given the historical preference for DBS in many neurological disorders (Levy et al., 1987), reassessing the potential role of ablative procedures in selected patient populations has become increasingly relevant.

The present study aimed to assess the efficacy of DBS and intracranial lesioning for the treatment of treatment-refractory chronic pain through a systematic review of the published literature, followed by a meta-analysis to quantitatively evaluate and compare trends in clinical pain outcomes. For this purpose, patients with cancer-related pain were excluded from the analysis because of the distinct pathophysiological mechanisms underlying oncological pain syndromes and their differing therapeutic expectations.

## 2 Materials and methods

### 2.1 Systematic literature search

**Fig. 1:**
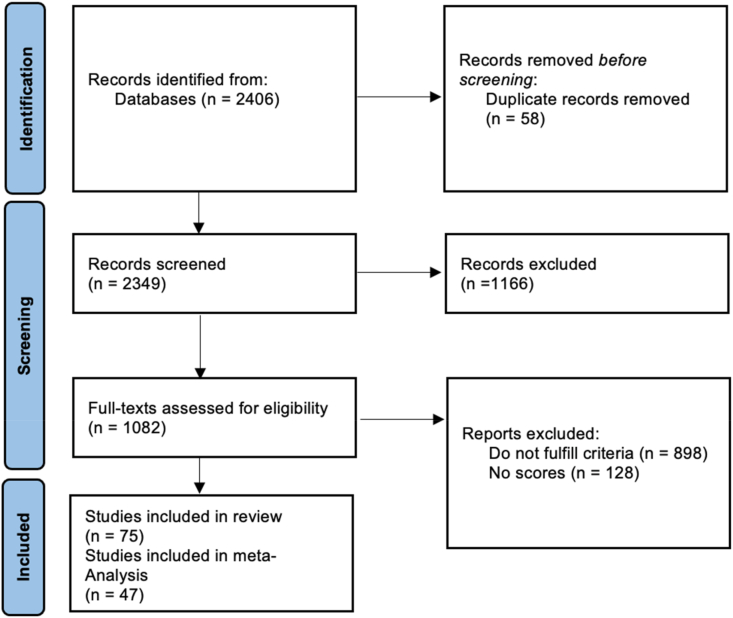
Adapted PRISMA 2020 flow diagram (Page et al., 2021).

A systematic literature search was conducted using the Preferred Reporting Items for Systematic Reviews and Meta-Analyses (PRISMA) guidelines (Page et al., 2021) (Fig. 1). PubMed was used as the electronic database to identify published literature on surgical treatments for treatment-refractory chronic pain. No filters were applied. The following search terms were used: “Deep brain stimulation AND chronic pain”, “Deep brain stimulation AND pain”; “Cingulotomy AND chronic pain”; “Cingulotomy AND pain”; “Thalamotomy AND chronic pain”; “Thalamotomy AND pain”; “Mesencephalotomy AND chronic pain”; and “Mesencephalotomy AND pain".

The literature search included all articles published between 1949 and April 2026. Studies were eligible for inclusion if they meet the following criteria: (1) case reports, case series, clinical trials, or randomized controlled studies of patients suffering from chronic treatment-refractory pain that underwent intracranial surgical procedures; (2) were original, published, and peer-reviewed; (3) written in English; (4) had a Digital Object Identifier (DOI).

Studies were excluded if (1) clinical data could not be extracted, (2) clinical data were not pain-specific, (3) clinical data were averaged across all patients when some patients did not meet the inclusion criteria (4) patients were already described in another article (in such cases, these patients were excluded; when this was not possible, the entire study was excluded), or (5) the pain was secondary to another disease, such as cancer-related pain.

The titles and abstracts of individual studies from the search results were independently reviewed for eligibility by two researchers (RS and RM-F).

### 2.2 Data extraction

The full texts of all screened articles were independently assessed by two researchers (RS and RM-F) for admissibility and compliance with the predefined selection criteria. When necessary, duplicate cases were identified by screening the demographic data available for the patients in the studies. The following variables were extracted from all studies included in the quantitative synthesis: number of participants, gender, age at surgery, target of surgery, duration of pain, pain source, location of pain, follow-up (FU) period, pre- and postsurgical data including the Visual Analog Scale (VAS), Numerical Rating Scale (NRS), Pain Intensity Score (PIS), Pain Rating Scale (PRS), Analog Scale (AS), Pain Relief (PR) or the Severity of Pain Score (SoPS), and percentage reduction of pain determined by a clinical score. Whenever possible, the data was collected per patient, otherwise averaged values were taken from all patients in the study and transferred to a predefined data extraction table. Finally, the extraction tables of both researchers were compared, and any discrepancies were resolved through re-evaluation of the original article.

If a patient underwent a repeated lesioning at certain period, the intervention was considered as an additional case. Furthermore, whenever sufficient information was available, pain syndromes were classified on a per-patient basis as neuropathic or nociceptive and additionally categorized as central vs peripheral pain syndromes. Face/head pain and phantom limb pain were analyzed as separate categories because these conditions are usually not assigned exclusively to a single category due to insufficient documentation or the presence of mixed pain phenotypes.

In addition, patients identified in the included studies were stratified according to surgical target whenever possible. For example, patients with two simultaneously active targets during the follow-up period were excluded from the target specific analysis. Table 1 reports the surgical targets according to the terminology used in the original publications. For DBS, targets were grouped into the following categories: anterior cingulate cortex, periventricular/periaqueductal gray, ventral striatum, internal capsule, lateral nuclei of the thalamus, ventral tegmental area, posterior hypothalamus, centro median thalamic nucleus, anterior thalamic nucleus and anterior pulvinar nucleus. Thalamotomy targets were also grouped into lateral thalamus and central thalamus.

**Table 1:** Overview of the included studies. N (number of patients in the studies); n (number of patients used from studies); DBS (Deep brain stimulation); NRS (Numeric Rating Scale); VAS (Visual Analogue Scale); PIS (pain intansity score); AS (analog scale); PRS (pain rating scale); SoPS (severity of pain Score); PR (pain relief); ACC (anterior cingulate cortex); PVA/PVG (periventricular/periaqueductal gray); NAC (nucleus accumbens); VPL (ventral posterolateral nucleus of thalamus); Vc (ventralis caudalis of thalamus); PLIC (posterior limb of the internal capsule); CmPF (thalamic centromedian parafascicular complex); VPM (ventral posteromedial nucleus of the thalamus); PAG (periaqueductal gray); VTA (ventral tegmental area); PVAG (periventricular gray/periaqueductal gray); ST (sensory thalamus); PH (posterior hypothalamic); PiH (posterior inferior hypothalamus); CMN (centromedian thalamic nucleus); VS (ventral striatum); ANT (anterior Thalamus nucleus); CM (centromedian nucleus); Pla (anterior pulvinar nucleus); IC (internal capsule); CLT (central lateral thalamotomy); CMT (central median thalamotomy); MT (median thalamotomy); NLM (nucleus lateralis mesencephali); Vcpci (nucleus ventrocaudalis parvocellularis internis); CM-Pf (centromedian and parafascicular Complex); VP (ventral posterior thalamus).

no.	Reference	Method	Evidence Level	N	n	Target	latest Follow-up	Scores
1	Abdallat et al., 2021 (Abdallat et al., 2021)	DBS	2	40	33	CmPf; VPL; VPM	11-180 M	VAS
2, 3	Abreu et al., 2017 (Abreu et al., 2017) Abreu et al., 2022 (Abreu et al., 2022)	DBS	3 3	16	16	VPL	Median: 60 M	VAS
4	Aibar-Durán et al., 2020 (Aibar-Duran et al., 2021)	DBS	2	7	7	PH	Median: 36 M	VAS
5	Akram et al., 2017 (Akram et al., 2017)	DBS	4	7	7	VTA	14-48 M	VAS
6	Alves and Asfora 2011 (Alves et al., 2011)	DBS	4	1	1	CMN	2 Wk	PIS
7	Basha et al., 2018 (Basha et al., 2018)	DBS	4	10	1	Vc; ST	1d	NRS
8	Ben-Haim et al., 2018 (Ben-Haim et al., 2018)	DBS	4	7	7	VPM; PAG	12 M	VAS
9	Bittar et al., 2005 (Bittar et al., 2005b)	DBS	4	3	3	PVG; VPL	20 M	VAS
10	Boccard et al., 2013 (Boccard et al., 2013)	DBS	2	97	38	PVG; VPL; VPM	12 M	VAS
11	Boccard et al., 2014 (Boccard et al., 2014a)	DBS	4	1	1	ACC	24 M	NRS
12	Boccard et al., 2014 (Boccard et al., 2014b)	DBS	3	16	3	ACC	6-17 M	VAS
13	Boccard et al., 2017 (Boccard et al., 2017)	DBS	3	11	11	ACC	12-60 M	NRS
14	Fontaine et al., 2025 (Fontaine et al., 2025)	DBS	3	8	8	ACC; ST	12 M	VAS
15	Franzini et al., 2020 (Franzini et al., 2020)	DBS	3	4	4	PLIC	20-28 M	NRS
16	Furlanetti et al., 2021 (Furlanetti et al., 2021)	DBS	4	1	1	VPL	4 Wk	VAS
17	Green et al., 2003 (Green et al., 2003)	DBS	4	1	1	VPL; PVG	6 M	VAS
18	Green et al., 2004 (Green et al., 2004)	DBS	3	7	7	VPL; PVG	6 M	VAS
19	Green et al., 2006a (Green et al., 2006a)	DBS	3	7	7	PVG; VPM	12 M	VAS
20	Green et al., 2006b (Green et al., 2006b)	DBS	3	11	11	PVG; VPL	12 M	VAS
21	Hamani et al., 2006 (Hamani et al., 2006)	DBS	4	13	5	Vc; PAG; PVG	12-108 M	VAS
22	Huang et al., 2016 (Huang et al., 2016)	DBS	4	10	10	PVA; PVG; VPL; VPM	6-12 M	VAS
23	Jermakowicz et al., 2017 (Jermakowicz et al., 2017)	DBS	4	1	1	PAG	13 M	NRS
24	Kashanian et al., 2020 (Kashanian et al., 2020)	DBS	4	9	7	VPM; PVG	40,3 M	VAS
25	Krüger et al., 2021 (Kruger et al., 2021)	DBS	4	1	1	VPM; CM; Pla	12 M	VAS
26	Kim et al., 2016 (Kim et al., 2016)	DBS	4	5	5	PAG; VPM; VPL	2-48 M	PRS
27	Lendvai and Kinfe 2017 (Lendvai et al., 2017)	DBS	4	1	1	ANT	72 M	VAS
28	Levi et al., 2019 (Levi et al., 2019)	DBS	3	9	5	ACC	5-42 M	NRS
29	Luo et al., 2020 (Luo et al., 2020)	DBS	3	12	12	PVA; PVG; ST	6-12 M	VAS
30	Mallory et al., 2012 (Mallory et al., 2012)	DBS	4	1	1	PVG; Vc; NAC	11 M	VAS
31	Mandat et al., 2023 (Mandat et al., 2023)	DBS	4	7	7	PAG; PVG	24 M	NRS
32	Morishita et al., 2015 (Morishita et al., 2015)	DBS	4	1	1	VS; Vc	12 M	AS
33	Nandi et al., 2003 (Nandi et al., 2003)	DBS	3	8	6	PVG	3-30 M	VAS
34	Niu et al., 2024 (Niu et al., 2024)	DBS	4	1	1	VPL; CLT	7 M	VAS
35	Owen et al., 2006 (Owen et al., 2006a)	DBS	3	26	23	PVG; Thalamus	1-44 M	VAS
36	Owen et al., 2006 (Owen et al., 2006b)	DBS	3	15	12	PVG; VPL	12 M	VAS
37	Pereira et al., 2007 (Pereira et al., 2007)	DBS	4	3	3	PVG; VPL	12 M	VAS
38	Pereira et al., 2013 (Pereira et al., 2013)	DBS	3	4	4	PVA; PVG	2 Wks	VAS
39	Piacentino et al., 2014 (Piacentino et al., 2014)	DBS	4	4	4	PH	60 M	PIS
40	Pickering et al., 2009 (Pickering et al., 2009)	DBS	4	1	1	PVG	6 Wks	NRS
41	Pinsker et al., 2008 (Pinsker et al., 2008)	DBS	4	2	1	PiH	3 M	AS
42	Polanski et al., 2019 (Polanski et al., 2019)	DBS	4	3	3	VPL; PVG; PLIC	12 M	NRS
43	Rezaei Haddad et al., 2015 (Rezaei Haddad et al., 2015)	DBS	4	1	1	Vcpci between VPL and VPM	38 M	PIS
44	Sims-Williams et al., 2016 (Sims-Williams et al., 2016)	DBS	4	3	3	PAG; CmPf	≥11 M	NRS
45	Son et al., 2014 (Son et al., 2014)	DBS	3	9	2	Vc	12-68 M	NRS
46	Tsubokawa et al., 1984 (Tsubokawa et al., 1984)	DBS	3	14	6	VPL; PAG; IC	approx. 2 Mo	SoPS
47	Wan et al., 2025 (Wan et al., 2025)	DBS	4	1	1	PAG; VP	12 M	VAS
48	Yamamoto et al., 2006 (Yamamoto et al., 2006)	DBS	3	18	18	Vc	12 M	VAS
**sum**	**48**	**DBS**		**438**	**312**			
49	Castillo Rangel et al., 2023 (Castillo Rangel et al., 2023)	Cingulotomy	3	6	6	ACC	6 M	VAS
50, 51	Cohen et al., 2001 (Cohen et al., 2001) Cohen et al., 1999 (Cohen et al., 1999)	Cingulotomy	3 3	12	12	ACC	12 M	PRS
52	Deng et al., 2018 (Deng et al., 2018)	Cingulotomy	4	1	1	ACC	24 M	NRS
53	Deng et al., 2019 (Deng et al., 2019)	Cingulotomy	4	2	2	ACC	18 M	NRS
54	Kollenburg et al., 2024 (Kollenburg et al., 2024)	Cingulotomy	4	1	1	ACC	3 Y	NRS
55	Pillay and Hassenbusch 1992 (Pillay et al., 1992)	Cingulotomy	4	10	2	cingulate gyrus	12 M	PRS
56	Sozer et al., 2025	Cingulotomy	4	1	1	ACC	12 M	NRS
57	Wang et al., 2017 (Wang et al., 2017)	Cingulotomy	3	24	24	ACC	6-77 M	VAS
58	Wilkinson et al., 1999 (Wilkinson et al., 1999)	Cingulotomy	3	23	13	ACC	1-15 Y	VAS
**sum**	**10**	**Cingulotomy**		**80**	**62**			
59	Ahmed et al., 2024 (Ahmed et al., 2024)	Thalamotomy	3	10	8	CLT	12 M	NRS
60	Franzini et al., 2021 (Franzini et al., 2021)	Thalamotomy	4	8	8	CLT	6-36 M	VAS
61	Franzini et al., 2023 (Franzini et al., 2023)	Thalamotomy	3	21	21	CLT	2-36 M	NRS
62	Gallay et al., 2020 (Gallay et al., 2020)	Thalamotomy	3	8	8	CLT	12-92 M	VAS
63	Gallay et al., 2023 (Gallay et al., 2023)	Thalamotomy	4	55	55	CLT	3-132 M	VAS
64	Hanuska et al., 2020 (Hanuska et al., 2020)	Thalamotomy	4	1	1	CMT (CM-Pf)	18 M	VAS
65	Ishaque et al., 2024 (Ishaque et al., 2024)	Thalamotomy	2	10	5	MT	1-3 M	NRS
66	Jeanmonod et al., 1993 (Jeanmonod et al., 1993)	Thalamotomy	3	45	39	MT	2 Wks - 38 M	VAS
67	Jeanmonod et al., 2012 (Jeanmonod et al., 2012)	Thalamotomy	3	11	11	CLT	3-12 M	VAS
68	Lara-Almunia et al., 2024 (Lara-Almunia et al., 2025)	Thalamotomy	4	9	8	CLT	37 M	VAS
69	Leal-Isaza et al., 2024 (Leal-Isaza et al., 2024)	Thalamotomy	4	50	50	CMT	38.5 M	VAS
70	Lovo et al., 2019 (Lovo et al., 2019)	Thalamotomy	3	14	10	CMT (CM-Pf)	1-33 M	VAS
71	Martin et al., 2009 (Martin et al., 2009)	Thalamotomy	3	9	9	CLT	2 d	PR
72	Michels et al., 2011 (Michels et al., 2011)	Thalamotomy	3	23	23	CLT	12 M	VAS
73	Pérez de la Perez de la Torre et al., 2021 (Perez de la Torre et al., 2021)	Thalamotomy	4	1	1	CMT	12 M	VAS
74	Urgosik and Liscak 2018 (Urgosik et al., 2018)	Thalamotomy	3	30	30	MT	12-180 M	PR
**sum**	**16**	**Thalamotomy**		**305**	**287**			
75	Liberson et al., 1970 (Liberson et al., 1970)	Mesen-cephalotomy	3	6	5	NLM	1d	PR
**sum**	**1**	**Mesen-cephalotomy**		**6**	**5**			

### 2.3 Study quality assessment

All included studies were independently assessed for possible bias by two independent researchers (RS and RM-F). The classification system proposed by French and Gronseth (French et al., 2008) was used since it provides a structured and pragmatic framework for evaluating levels of evidence in neurosurgical research, particularly in fields with limited availability of randomized controlled trails. This system compromises four different levels of evidence, with level 1 corresponding to a high-quality study with a low risk of bias. This risk of bias progressively increases across levels 2 and 3, whereas level 4 represents studies with a high risk of bias (Table 1).

### 2.4 Statistical analysis

All clinical scores included in this study (VAS, NRS, PIS, AS, PRS or SoPS) were designed to quantify pain severity. On these scales, a value of zero represents no pain, whereas increasing values indicate increasing pain intensity, with the maximum score, 10 or 100, representing the highest amount of intolerable pain. Clinical outcomes were analyzed across predefined time points: preoperative baseline (T0) and postoperative time points T1 (≤1 month), T2 (>1 month - ≤ 6 months), T3 (>6 months - ≤ 12 months) and T4 (>12 months). In some studies, the absolute scores of patients were available, which were extracted to calculate the mean score per time point within the study. The mean value was calculated with the last clinical score within that period when; data for a given timepoint was reported fully, only a single patient was available within the specified interval, or multiple assessments for the same patient were reported within a single time interval. In addition, two studies reported only median values, which were subsequently used for further calculations. After the mean values were calculated, the Percentage Pain relief (P%) from baseline to T1-T4 was calculated (Table 2). For this purpose, the simple formula was used here using the example of the P% at T1:

P% = 100 – ((⌀ T1 / T0) ∗ 100)

**Table 2:** Mean percent pain reduction per study. For T0, the mean baseline score or a ratio is reported where available; otherwise, it is marked as n.a. (not available). Median baseline score is reported in two cases. For T1 to T4 the mean percent pain reduction is given by study.

no.	Reference	Method	mean	P%			
			T0	T1 (≤1M)	T2 (>1M -≤ 6M)	T3 (>6M - ≤ 12 M)	T4 (>12M)
1	Abdallat et al., 2021 (Abdallat et al., 2021)	DBS	6,94	44,54%	44,54%	45,24%	45,24%
2, 3	Abreu et al., 2017 (Abreu et al., 2017) Abreu et al., 2022 (Abreu et al., 2022)	DBS	median: 9	-	-	61,11%	77,78%
4	Aibar-Durán et al., 2020 (Aibar-Duran et al., 2021)	DBS	median: 8	-	50,00%	62,50%	50,00%
5	Akram et al., 2017 (Akram et al., 2017)	DBS	8,57	-	-	-	46,67%
6	Alves and Asfora 2011 (Alves et al., 2011)	DBS	10	80%	-	-	-
7	Basha et al., 2018 (Basha et al., 2018)	DBS	7	64,28%	-	-	-
8	Ben-Haim et al., 2018 (Ben-Haim et al., 2018)	DBS	9	-	-	71,43%	-
9	Bittar et al., 2005 (Bittar et al., 2005b)	DBS	4,33	-	-	58,00%	70,00%
10	Boccard et al., 2013 (Boccard et al., 2013)	DBS	7,9	-	32,91%	37,97%	-
11	Boccard et al., 2014 (Boccard et al., 2014a)	DBS	6,7	-	-	40,30%	55,22%
12	Boccard et al., 2014 (Boccard et al., 2014b)	DBS	8,1	87,65%	50,62%	62,96%	100,00%
13	Boccard et al., 2017 (Boccard et al., 2017)	DBS	8,04	-	53,96%	39,34%	6,05%
14	Fontaine et al., 2025 (Fontaine et al., 2025)	DBS	7,06	-	-	35,42%	-
15	Franzini et al., 2020 (Franzini et al., 2020)	DBS	9	66,67%	-	-	38,89%
16	Furlanetti et al., 2021 (Furlanetti et al., 2021)	DBS	7-9	50%	-	-	-
17	Green et al., 2003 (Green et al., 2003)	DBS	6,9	-	100,00%	-	-
18	Green et al., 2004 (Green et al., 2004)	DBS	8,11	-	41,55%	-	-
19	Green et al., 2006a (Green et al., 2006a)	DBS	7,96	-	-	53,32%	-
20	Green et al., 2006b (Green et al., 2006b)	DBS	n.a.	-	-	45,82%	-
21	Hamani et al., 2006 (Hamani et al., 2006)	DBS	8,8	-	-	54,55%	63,07%
22	Huang et al., 2016 (Huang et al., 2016)	DBS	7,48	-	29,81%	19,28%	-
23	Jermakowicz et al., 2017 (Jermakowicz et al., 2017)	DBS	8	0,00%	75,00%	62,50%	75,00%
24	Kashanian et al., 2020 (Kashanian et al., 2020)	DBS	9,4	-	-	-	35,11%
25	Krüger et al., 2021 (Kruger et al., 2021)	DBS	7,5	-	93,33%	93,33%	-
26	Kim et al., 2016 (Kim et al., 2016)	DBS	n.a.	-	70,00%	-	30,00%
27	Lendvai and Kinfe 2017 (Lendvai et al., 2017)	DBS	8	56,25%	-	-	-
28	Levi et al., 2019 (Levi et al., 2019)	DBS	7,6	-	55,26%	38,60%	14,47%
29	Luo et al., 2020 (Luo et al., 2020)	DBS	7,74	-	-	24,87%	-
30	Mallory et al., 2012 (Mallory et al., 2012)	DBS	10	80,00%	90,00%	100,00%	-
31	Mandat et al., 2023 (Mandat et al., 2023)	DBS	8,57	-	55,00%	48,33%	45,00%
32	Morishita et al., 2015 (Morishita et al., 2015)	DBS	8-9	-	-	0,00%	-
33	Nandi et al., 2003 (Nandi et al., 2003)	DBS	n.a.	-	36,00%	41,50%	46,00%
34	Niu et al., 2024 (Niu et al., 2024)	DBS	8	-	12,50%	37,50%	-
35	Owen et al., 2006 (Owen et al., 2006a)	DBS	8,12	50,29%	50,29%	50,29%	50,29%
36	Owen et al., 2006 (Owen et al., 2006b)	DBS	8,1	-	-	48,80%	-
37	Pereira et al., 2007 (Pereira et al., 2007)	DBS	9,03	-	-	38,01%	-
38	Pereira et al., 2013 (Pereira et al., 2013)	DBS	8,58	63,25%	-	-	-
39	Piacentino et al., 2014 (Piacentino et al., 2014)	DBS	9,25	-	-	-	64,86%
40	Pickering et al., 2009 (Pickering et al., 2009)	DBS	10	60,00%	-	-	-
41	Pinsker et al., 2008 (Pinsker et al., 2008)	DBS	10	-	10,00%	-	-
42	Polanski et al., 2019 (Polanski et al., 2019)	DBS	6,67	-	40,00%	55,00%	-
43	Rezaei Haddad et al., 2015 (Rezaei Haddad et al., 2015)	DBS	10	-	-	-	40%
44	Sims-Williams et al., 2016 (Sims-Williams et al., 2016)	DBS	9,17	81,82%	-	72,73%	-
45	Son et al., 2014 (Son et al., 2014)	DBS	8	-	-	-	37,50%
46	Tsubokawa et al., 1984 (Tsubokawa et al., 1984)	DBS	12,33	-	82,33%	-	-
47	Wan et al., 2025 (Wan et al., 2025)	DBS	7	-	-	85,71%	-
48	Yamamoto et al., 2006 (Yamamoto et al., 2006)	DBS	n.a.	-	-	66,40%	
49	Castillo Rangel et al., 2023 (Castillo Rangel et al., 2023)	Cingulotomy	9,33	82,14%	82,14%	-	-
50, 51	Cohen et al., 2001 (Cohen et al., 2001) Cohen et al., 1999 (Cohen et al., 1999)	Cingulotomy	8,6	-	-	20,93%	-
52	Deng et al., 2018 (Deng et al., 2018)	Cingulotomy	7	-	-	71,43%	85,71%
53	Deng et al., 2019 (Deng et al., 2019)	Cingulotomy	9,5	-	42,11%	68,42%	78,95%
54	Kollenburg et al., 2024 (Kollenburg et al., 2024)	Cingulotomy	9	-	44,44%	100%	100%
55	Pillay and Hassenbusch 1992 (Pillay et al., 1992)	Cingulotomy	8,5	-	-	29,41%	-
56	Sozer et al., 2025	Cingulotomy	9	-	88,89%	72,22%	-
57	Wang et al., 2017 (Wang et al., 2017)	Cingulotomy	7,75	61,29%	35,48%	-	35,48%
58	Wilkinson et al., 1999 (Wilkinson et al., 1999)	Cingulotomy	7-10	40%	-	-	-
59	Ahmed et al., 2024 (Ahmed et al., 2024)	Thalamotomy	7,2	-	-	44,44%	-
60	Franzini et al., 2021 (Franzini et al., 2021)	Thalamotomy	9,38	-	55,60%	33,30%	36,00%
61	Franzini et al., 2023 (Franzini et al., 2023)	Thalamotomy	9	-	23,28%	23,28%	23,28%
62	Gallay et al., 2020 (Gallay et al., 2020)	Thalamotomy	5,03	-	51,25%	71,25%	74,29%
63	Gallay et al., 2023 (Gallay et al., 2023)	Thalamotomy	5,4	40,74%	-	-	-
64	Hanuska et al., 2020 (Hanuska et al., 2020)	Thalamotomy	9-10	100,00%	-	-	68,42%
65	Ishaque et al., 2024 (Ishaque et al., 2024)	Thalamotomy	6,4	21,88%	−3,12%	-	-
66	Jeanmonod et al., 1993 (Jeanmonod et al., 1993)	Thalamotomy	6	53,33%	53,33%	53,33%	53,33%
67	Jeanmonod et al., 2012 (Jeanmonod et al., 2012)	Thalamotomy	5,95	71,10%	49,40%	56,90%	-
68	Lara-Almunia et al., 2024 (Lara-Almunia et al., 2025)	Thalamotomy	9	-	-	52,78%	38,10%
69	Leal-Isaza et al., 2024 (Leal-Isaza et al., 2024)	Thalamotomy	10	-	-	-	40,00%
70	Lovo et al., 2019 (Lovo et al., 2019)	Thalamotomy	10	100,00%	-	34,00%	45,00%
71	Martin et al., 2009 (Martin et al., 2009)	Thalamotomy	n.a.	68,00%	-	-	-
72	Michels et al., 2011 (Michels et al., 2011)	Thalamotomy	6,42	-	-	37,40%	-
73	Pérez de la Perez de la Torre et al., 2021 (Perez de la Torre et al., 2021)	Thalamotomy	9-10	-	-	78,95%	-
74	Urgosik and Liscak 2018 (Urgosik et al., 2018)	Thalamotomy	n.a.	-	-	-	69,17%
75	Liberson et al., 1970 (Liberson et al., 1970)	Mesen-cephalotomy	n.a.	100,00%	-	-	-

**Fig. 2:**
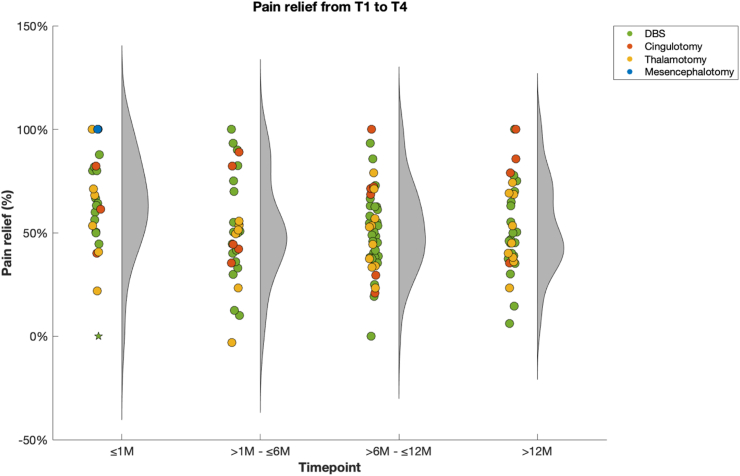
Violin plots of mean percent pain reduction per method and study for each time point. The stars represent the outliers and colors indicate the corresponding methods.

**Fig. 3:**
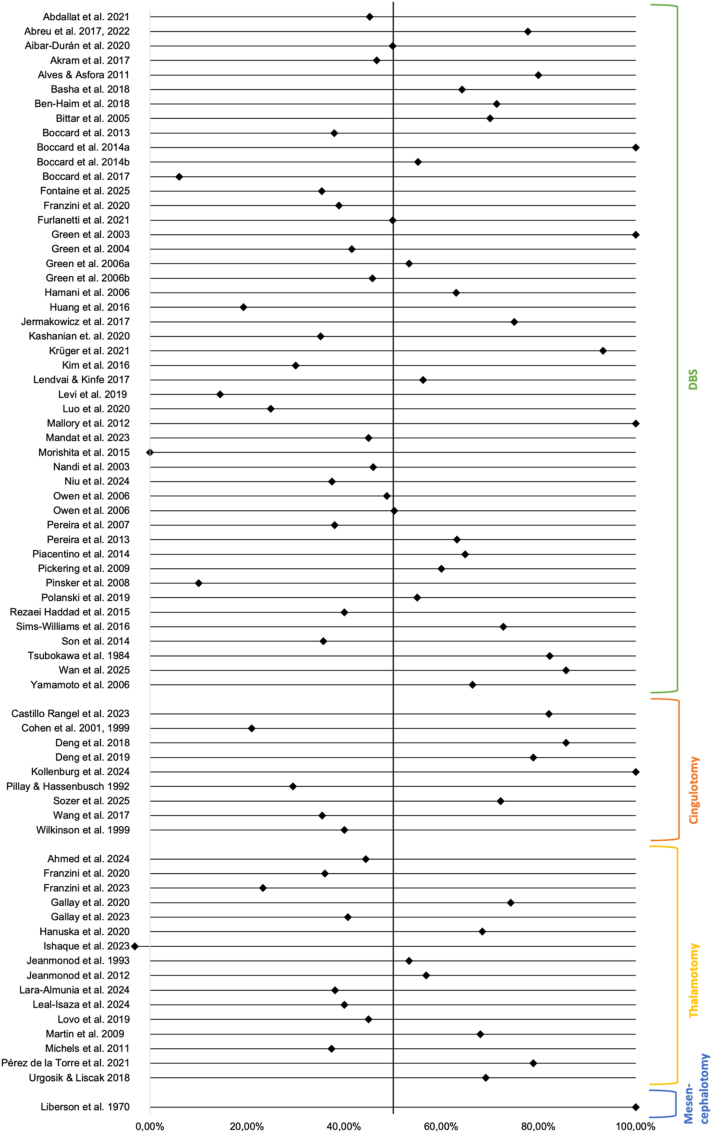
Forest plot showing the mean percent reduction of each selected study at the last follow up. The diamond shows the scores and the color code indicates the corresponding method. Note that the middle line denotes a relief of 50% of the pain score.

If the P% was already reported on a per-patient basis, the mean value for each timepoint was calculated. The number of patients available could vary between time points. When multiple P% were available for the same patient within a timepoint, the last one within the time point was included. Results were analyzed separately for each surgical procedure. Additionally, P% was calculated for the categorical variables neuropathic versus nociceptive pain, central versus peripheral pain syndromes, as well as phantom limb and head/face pain. Furthermore, P% values were calculated for the respective anatomical targets in DBS and thalamotomy. In some cases, the P% had already been reported for the entire cohort and was extracted directly (Fig. 2, Fig. 3).

### 2.5 Statistical analysis for the meta-analysis

**Fig. 4:**
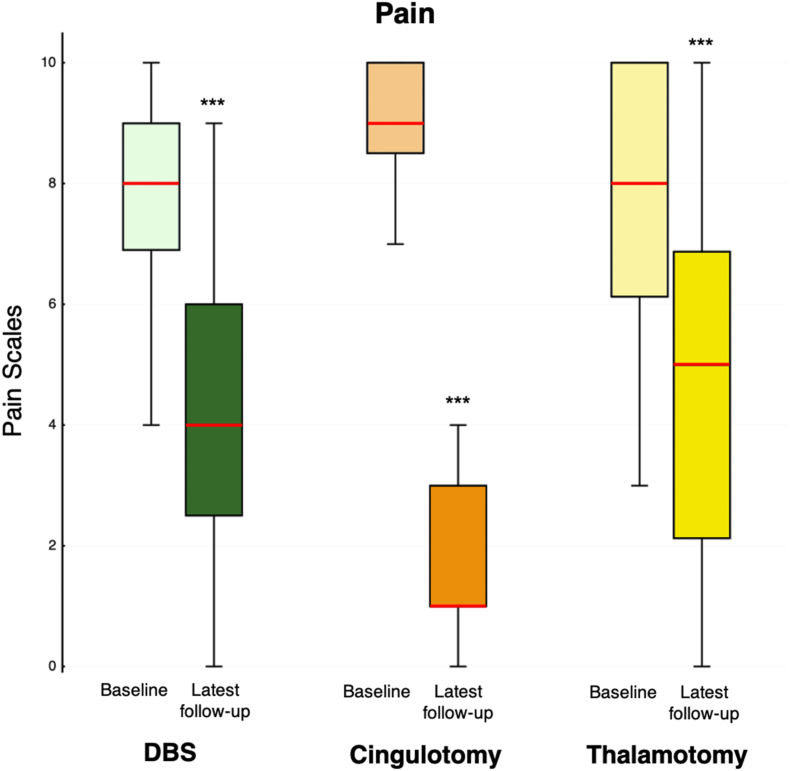
The total pain scores change between baseline and latest follow up for different methods. The color code indicates the surgical method. Light colors represent baseline, while darker colors represent the latest follow-up. The boxes show the lower and upper quartile, the median and the whiskers represent the minimum and maximum of the absolute patient's pain scores used in the meta-analysis. ∗∗∗p < 0.001.

A meta-analysis was performed using data from the studies that reported both baseline and last follow-up scores for each included patient. These studies were categorized according to treatment modality: DBS, cingulotomy and thalamotomy. A Wilcoxon signed rank test was applied to compare the baseline pain scores with the last reported score within each procedure. Significant differences were identified among all three treatment groups. To quantify the magnitude of these differences while minimizing potential sample size bias, the effect sizes calculated using Hedges’ g. Values ≥ 0.8 were considered indicative of a large effect size (Fig. 4).

**Fig. 5:**
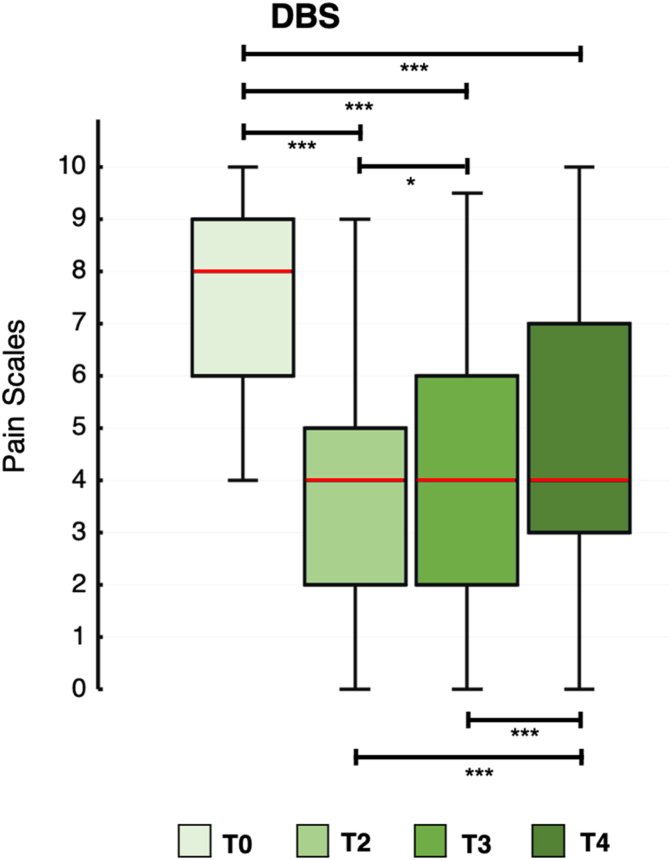
Pain score change between baseline (T0) and T2 to T4 for DBS. The boxes show the lower and upper quartile, the median and the whiskers represent the minimum and maximum of the absolute patient's pain scores used in the meta-analysis for DBS at the different timepoints. ∗p < 0.05; ∗∗∗p < 0.001.

DBS was the only treatment modality for which sufficient repeated outcome measurements were available across multiple follow-up intervals. Due to the heterogeneity of the available data and the limited number of observations, insufficient data were available to include T1 in the statistical analysis. Patients with assessments at T0 (Baseline), T2 (>1 M - ≤ 6 M), T3 (>6 M - ≤ 12 M) and T4 (>12 M) were included for this analysis. A Friedman test was performed to assess differences across different time points, followed by a Nemenyi post-hoc test to search for the specific differences among the clinical results (Fig. 5). All statistical analyses were performed using SPSS 31.0 for Mac (SPSS, Inc., Chicago, IL, USA). Statistical significance was defined as α = 0.05 with β = 0.2.

## 3 Results

### 3.1 Study selection

A PubMed search retrieved 2406 articles investigating intracranial surgical procedures for treatment-refractory chronic pain. After removal of duplicates and application of the predefined inclusion criteria, 75 studies were included, of which 47 provided extractable pain scores for meta-analysis. The included studies comprised DBS (n = 48), cingulotomy (n = 10), thalamotomy (n = 16), and mesencephalotomy (n = 1). The study selection process is summarized in the PRISMA flowchart (Fig. 1).

### 3.2 Individual participant data

From the 75 included studies, a total of 666 patients were included in the final analysis (Table 1). The majority of patients were male with 61.26%. The mean age at surgery was 54 years (range: 21-89 years). From the 666 included patients, 312 (46.85%) underwent DBS, 62 (9,31%) underwent cingulotomy, 287 (43.09%) underwent thalamotomy, and only 5 (0.75%) underwent mesencephalotomy.

### 3.3 Clinical outcomes analysis

Pain outcomes reported from baseline to the predefined postoperative time points are summarized in Table 2 and Fig. 2. All studies excepted one demonstrated a reduction in pain severity. One thalamotomy study reported on a worsening of symptoms at time point two, corresponding to a pain reduction of 3.12% (Table 2 and Fig. 3).

The distribution of studies reporting a P% ≥ 50% was as follows: DBS T1 (10/13), T2 (11/20), T3 (14/29), T4 (7/19); cingulotomy T1 (2/3), T2 (2/5), T3 (4/6), T4 (3/4); thalamotomy T1 (5/7), T2 (3/6), T3 (5/10), T4 (4/9); and mesencephalotomy T1 (1/1). The follow-up duration ranged from immediately after the intervention until up to 15 years FU (Table 1). From all included studies, the mean duration of the last follow-up was 2.68 years (SD: 3.35 years).

From the 75 included studies, 66 provided sufficient patient level data to allow categorization according to pain etiology across all procedures and target specific analyses for DBS and thalamotomy.

Within the pain etiology categories, no useable patient level data were available for mesencephalotomy. The remaining surgical procedures predominantly included patients with neuropathic pain syndromes, whereas clearly identifiable nociceptive pain syndromes were largely absent. Patients with a mixed phenotype were excluded from this categorization.

In the neuropathic pain group, the mean P% was 44.81% for DBS, 64.43% for cingulotomy and 51.21% for thalamotomy. In addition, patients with cluster headache who underwent DBS demonstrated a mean P% of 47.76% (Table 3).

**Table 3:** Mean percent pain reduction by pain etiology and surgical procedure. For each subgroup, the number of patients (n), mean percent pain reduction (mean P%), and standard deviation (SD P%) are reported where available. “Pats all” indicates the total number of patients per pain category, while “Pat % of 666” represents the proportion of the total study population. Missing or unavailable data are marked with “-“.

Neuropathic vs. Nociceptive														
	DBS			Cingulotomy			Thalamotomy			Mesencephalotomy			Pats all	Pat % of 666
	n	mean P%	SD P%	n	mean P%	SD P%	n	mean P%	SD P%	n	mean P%	SD P%		
**Neuropathic**	231	44,81	31,33	31	64,43	29,72	269	51,21	37,04	0	-	-	531	79,73
**Nociceptive**	0	-	-	0	-	-	0	-	-	0	-	-	0	0,00
**Cluster headache**	19	47,76	33,66	0	-	-	0	-	-	0	-	-	19	2,85
**SUM**													550	

Head/Facial														
	DBS			Cingulotomy			Thalamotomy			Mesencephalotomy			Pats all	Pat % of 666
	n	mean P%	SD P%	n	mean P%	SD P%	n	mean P%	SD P%	n	mean P%	SD P%		
**Trigeminal neuralgia**	11	54,39	31,16	0	-	-	40	61,95	38,38	0	-	-	51	7,66
**Cluster headache**	19	47,76	33,66	0	-	-	0	-	-	0	-	-	19	2,85
**Others**	13	56,77	25,43	0	-	-	1	100,00	-	0	-	-	14	2,10
**SUM**													84	

Central vs. Peripheral														
	DBS			Cingulotomy			Thalamotomy			Mesencephalotomy			Pats all	Pat % of 666
	n	mean P%	SD P%	n	mean P%	SD P%	n	mean P%	SD P%	n	mean P%	SD P%		
**Central**	79	38,26	33,83	2	55,56	62,85	21	58,95	37,70	0	-	-	102	15,32
**Peripheral**	43	46,28	32,16	1	50,00	-	111	50,47	39,51	0	-	-	155	23,27
**Phantom limb**	20	57,49	26,52	1	85,71	-	2	30,00	28,28	0	-	-	23	3,45
**SUM**													280	

Regarding the analysis of head and facial pain identified eligible data for DBS and thalamotomy. Among patients with trigeminal neuralgia, the mean P% was 54.39% following DBS and 61.95% following thalamotomy. The cluster headache subgroup remained unchanged. Within the category “others”, patients treated with DBS achieved a mean P% of 56.77%, whereas the single patient treated with thalamotomy patient achieved complete pain reduction (Table 3).

For the classification analysis of central versus peripheral pain, no suitable data were available for mesencephalotomy. Among patients with central pain syndromes, the mean P% was 38.26% for DBS, 55.56% for cingulotomy and 58.95% for thalamotomy. Among patients with peripheral pain syndromes, the mean P% was 46.28% for DBS, 50% for cingulotomy and 50.47% for thalamotomy. Phantom limb pain was analyzed separately. The mean P% was 57.49% for DBS, 85.71% for cingulotomy and 30% for thalamotomy (Table 3).

The target-based analyses for DBS and thalamotomy are summarized in Table 4. Within the DBS cohort, the most frequently used targets were the periventricular/periaqueductal gray (n = 63; P% 50.85% SD: 24.80) and the lateral nuclei of the thalamus (n = 65; P% 55.45% SD: 25.64%), followed by the anterior cingulate cortex (n = 20; P% 15.26% SD: 36.77) and the centro median thalamic nucleus (n = 20; P% 47.04% SD: 46.05). No patients were identified with ventral striatum and anterior pulvinar nucleus as isolated stimulation targets (Table 4).

**Table 4:** Mean percent pain reduction by target and surgical procedure. For each subgroup, the number of patients (n), mean percent pain reduction (mean P%), and standard deviation (SD P%) are reported where available.

DBS Targets			
	n	mean P%	SD P%
**anterior cingulate cortex**	20	15,26	36,77
**periventricular/periaqueductal gray**	63	50,85	24,80
**internal capsule**	6	49,58	29,63
**lateral nuclei of the thalamus**	65	55,45	25,64
**ventral tegmental area**	7	42,54	40,48
**posterior hypothalamus**	12	53,84	25,88
**centro median thalamic nucleus**	20	47,04	46,05
**anterior thalamic nucleus**	1	56,25	-

Thalamotomy Targets			
	n	mean P%	SD P%
**lateral thalamus**	133	44,97	36,12
**central thalamus**	136	59,12	37,04

Regarding the thalamotomy cohort, lateral and central thalamus were targeted with comparable frequency. However, procedures targeting the central thalamus were associated with approximately 10% grater mean pain reduction than lateral thalamotomy (Table 4).

### 3.4 Meta-analysis

Consistent with the descriptive analyses, all surgical procedures demonstrated clinically meaningful pain reduction (Bittar et al., 2005a; Pereira et al., 2013). Due to the non-normal distribution of the data, Hedges’ g was used to quantify the effect size on pain scores between baseline and last follow-up for all included treatment modalities (DBS: p < 0.001, g = 1.44 85-90%; cingulotomy: p < 0.001, g = 2.6 > 95%; thalamotomy: p < 0.001, g = 0.92 ≈ 74%). These results showed that all three methods had a significant pain reduction when comparing preoperative and postoperative scores at maximum follow-up (Fig. 4). Despite these overall findings, intraindividual patient analysis demonstrated that some patients experienced little or no clinical benefit, with comparable pain scores at baseline and maximum follow-up (DBS: 5,2%; cingulotomy: 0%, thalamotomy: 15%).

Only a subset of the included DBS studies reported individual patients’ pain data across multiple time points, allowing a longitudinal analysis. Consequently, 47 studies were included in the time course analysis. A Friedman test followed by a post-hoc Nemenyi test was performed to quantify the results (Fig. 5). Significant differences were observed across all evaluated time points (∗∗∗ = p < 0.001, ∗ = p < 0.05).

## 4 Discussion

This study demonstrates that surgical intracranial procedures, including both ablative and neuromodulatory techniques, effectively reduce pain in patients with chronic treatment-refractory pain at long-term follow-up. Significant pain reduction was observed across all surgical approaches, particularly following DBS, cingulotomy, and thalamotomy. Although pain levels in the DBS cohort tended to increase again over time, they remained significantly lower than the preoperative baselines throughout follow-up periods. As DBS is currently more widely adopted because of its adaptability and potential reversibility, the number of patients treated with DBS was substantially greater than that of patients undergoing ablative procedures.

Similarly, DBS was the most extensively investigated surgical modality, accounting for the largest number of studies included in our analysis. Moreover, follow-up reporting was more consistent for DBS than for the other surgical approaches. In contrast, mesencephalotomy was the least represented intervention, with only a single published study available. Although this study reported complete pain relief at the first follow-up time point (T1), mesencephalotomy was excluded from the meta-analysis because of insufficient short-term data. Across all surgical modalities, only a limited number of studies reported complete pain resolution from baseline to the final follow-up assessment. These findings suggest that surgical interventions for pain management are unlikely to achieve total pain elimination and should instead be regarded as strategies aimed at reducing symptom burden and improving patients' quality of life.

Neuropathic pain syndromes predominated across all surgical interventions, whereas clearly defined nociceptive pain syndromes were largely absent. Cluster headache was exclusively represented in the DBS cohort. Although cingulotomy comprised the smallest patient cohort, it was associated with the greatest mean reduction in pain severity. In contrast, the DBS and thalamotomy cohorts were substantially larger, despite demonstrating pain reductions that were up to 10% lower. Across all modalities, large standard deviations (SD: 27-29%) indicated considerable heterogeneity in treatment response. Regarding head and facial pain syndromes, DBS was applied to trigeminal neuralgia, cluster headache, and other pain conditions in relatively similar proportions, whereas thalamotomy was used predominantly for trigeminal neuralgia. Treatment outcomes within these subgroups were also highly variable (SD: 25%-38%). Within the categories of central and peripheral pain syndromes, only a small number of patients underwent cingulotomy, whereas DBS and thalamotomy accounted for the majority of cases. DBS was more frequently employed for central pain syndromes, while thalamotomy was more commonly used for peripheral pain syndromes. Phantom limb pain represented only a small proportion of cases in both groups. Reported pain reduction ranged from 38% to 57% following DBS and from 30% to 58% following thalamotomy, further underscoring the substantial underlying heterogeneity in treatment response.

The target-based analysis showed that the periventricular/periaqueductal gray and the lateral thalamic nuclei were the most frequently selected DBS targets, reflecting their established roles within pain modulation networks. However, the large standard deviations observed across target groups indicate substantial variability in treatment outcomes, underscoring the importance of individualized target selection. In thalamotomy, interventions targeting the central thalamus were associated with slightly greater improvements than those directed at lateral thalamic regions. Overall, these findings highlight the need for more standardized target selection strategies to reduce heterogeneity in clinical outcomes and improve comparability across future studies.

Our meta-analysis included three surgical modalities, all of which demonstrated statistically significant improvements from baseline to the latest follow-up assessment. These findings support the role of DBS, cingulotomy, and thalamotomy as viable long-term treatment options, with the choice of intervention guided by individualized clinical assessment. For DBS, comparisons between baseline and the different follow-up time points revealed significant reductions in pain scores. The only exception was the comparison between T2 and T3, which did not reach statistical significance. Across all three surgical modalities, the greatest improvement was observed at T1, a finding that may, at least partially, be explained by the well-described microlesion or stun effect (Nishiguchi et al., 2022). The subsequent difference between T1 and T2 may reflect the gradual resolution of this initial effect, together with the time required to optimize stimulation parameters. In contrast, no significant differences were observed between T2 and T3. However, the significant difference between T3 and T4 underscores the importance of long-term follow-up extending beyond one year. Although cingulotomy was associated with the greatest reduction in pain scores between baseline and the latest follow-up, this finding should be interpreted with caution given the small cohort size. Overall, these results further emphasize the substantial heterogeneity in treatment response across surgical techniques and highlight the need for more standardized patient selection criteria and treatment strategies.

DBS was the most extensively investigated treatment modality in the available literature. Consequently, a wide range of stimulation targets was identified, reflecting both patient-specific clinical needs and surgeons’ expertise (ACC, PVG, Vc, NAC, VPL, PLIC, CmPf, PAG, VPM, VTA, ST, PH, Vcpic, PiH, CMN, VS, ANT, Pla, and IC). One of the principal advantages of DBS is its adaptability, as stimulation parameters can be adjusted over time to optimize therapeutic outcomes. Accordingly, regular and well-documented follow-up assessments are essential to maximize long-term clinical benefit. However, DBS also carries the risk of stimulation habituation and a gradual decline in efficacy over time. In patients with treatment-refractory chronic pain who have exhausted the potential for further stimulation optimization, ablative procedures may still represent a valuable therapeutic alternative.

The main limitation of this systematic review was the substantial heterogeneity among the included studies, particularly regarding pain assessment methods and follow-up duration, which ranged from the immediate postoperative period to a maximum of 180 months. In addition, the literature search was restricted to the PubMed database and may therefore have missed relevant studies indexed elsewhere. Consequently, a longitudinal meta-analysis could only be performed for patients who underwent DBS. To avoid further fragmentation of the neuromodulation cohort, different DBS targets were not analyzed separately. Consistent with this inclusive approach, studies with lower levels of evidence, including case reports, were incorporated, thereby increasing the overall risk of bias. Although the French and Gronseth (French et al., 2008) classification system provides a practical framework for evaluating heterogeneous evidence, it is less comprehensive than more widely adopted contemporary risk-of-bias assessment tools. Mesencephalotomy was excluded from treatment comparisons because individual patient data were unavailable. Likewise, the earliest postoperative follow-up time point (T1) could not be included in most analyses because these data were not consistently reported. Furthermore, many studies reported only aggregated outcomes rather than individual patient data, limiting patient-level comparisons and reducing the sample size available for meta-analysis. Additional studies had to be excluded because the clinical scales used to assess pain outcomes were not clearly specified. Future research should therefore adopt standardized and clearly defined outcome measures. Establishing a core outcome set would facilitate more consistent reporting, improve comparability across studies, and enable more robust evaluations of different surgical treatment modalities.

The interpretation of treatment effects following intracranial surgical interventions for chronic pain may be influenced by placebo and other contextual effects. As many of the included studies lacked controlled study designs, distinguishing true therapeutic benefits from placebo responses remains challenging. Future investigations would benefit from more rigorous methodological approaches, including randomized controlled trials and blinded ON/OFF stimulation paradigms for DBS, to better assess treatment specificity. During the systematic literature search, several studies employing ON/OFF stimulation protocols were identified; however, they did not meet the remaining inclusion criteria and were therefore excluded from the present analysis (Owen et al., 2007; Pereira et al., 2010; Ray et al., 2009). These limitations should be considered when developing future protocols for invasive intracranial procedures to ensure that surgical decision-making remains evidence-based and ethically sound.

Despite the demonstrated efficacy of several surgical approaches for chronic pain, important challenges remain before consistent outcomes can be achieved across homogeneous patient cohorts and different pain indications. Emerging technologies may help address these limitations. Advances in connectivity-based neuroimaging could improve the identification of optimal surgical targets and facilitate the optimization of stimulation parameters in patients undergoing DBS. In addition, non-invasive neuromodulation techniques, such as transcranial magnetic stimulation, transcranial direct current stimulation, and transcranial pulse stimulation, may represent alternative or adjunctive therapeutic options to intracranial surgical interventions. Similarly, minimally invasive lesioning techniques, including laser interstitial thermal therapy and high-intensity focused ultrasound, remain insufficiently investigated. Nevertheless, these approaches show considerable promise for the future of surgical pain management.

## 5 Conclusion

This review with meta-analysis highlights that multiple surgical approaches for chronic pain are effective, with all methods demonstrating significant pain reduction. Importantly, these treatments should be viewed not as curative but as strategies to achieve meaningful symptom relief. Future studies should include standardized pain scales, consistent follow-up time points, and quantitative reporting to reduce bias and strengthen the evidence base.

## Contributions

Methodology: RS, PH, VVV, PA. Data extraction: RS, RMF. Validation of the Study: RS, RMF, PA. Data curation: RS, FXCR, RL. Statistical analysis: FXCR. Coding: RS, RL. Writing of the manuscript: RS, FXCR, PA. Review of the manuscript: VVV, RL, PH, RMF. Supervision: PA Project administration: VVV, PA.

## Funding

The authors acknowledge financial support provided by 10.13039/501100012681University Hospital Cologne for this study.
